# Identifying dyslexia-consistent reading profiles in mild intellectual disability: cluster-derived severity gradients and severity-calibrated classification rules

**DOI:** 10.3389/fpsyt.2026.1805069

**Published:** 2026-05-29

**Authors:** Bartosz M. Radtke, Paweł Jurek, Michał Olech, Ariadna Łada-Maśko, Urszula Sajewicz-Radtke

**Affiliations:** 1 Laboratory of Psychological and Educational Tests, Gdańsk, Poland; 2 Institute of Psychology, University of Gdańsk, Gdańsk, Poland; 3 Department of Psychology, Medical University of Gdańsk, Gdańsk, Poland

**Keywords:** decoding, developmental learning disorder, intellectual disability, phonological processing, RAN, reading fluency

## Abstract

**Introduction:**

Translating dyslexia constructs to mild intellectual disability (MID) is challenging because population-based cut-offs may label broadly expected low attainment as ‘specific’. This study combined person-centered profiling with explicit, ICD-11–guided, severity-calibrated classification rules to quantify when and why over-identification occurs in MID.

**Methods:**

Nationwide diagnostic data from 446 students with MID were analyzed using age-normed sten scores from the SB6/18 battery (decoding, reading fluency, reading comprehension, phonetic coding, rapid automatized naming [RAN]). We conducted hierarchical clustering with bootstrap stability and compared six rule-based groupings that systematically varied severity thresholds and inclusion of phonological/RAN indicators.

**Results:**

A robust two-cluster solution reflected a dominant severity gradient across reading and related markers. Broad criteria classified 88% of the sample, including many higher-performing cases. Introducing an extreme-severity anchor reduced prevalence (37%) and concentrated classified cases within the lower-performing profile. Notably, when very low decoding (sten = 1) was required, adding phonological/RAN criteria yielded little further classification gain under this rule structure.

**Conclusions:**

Dyslexia-consistent profiles can be delineated within mild ID, but prevalence and subgroup coherence depend critically on severity operationalization. Word-level reading—especially decoding and fluency—provides the most robust basis for subgroup identification; phonological/RAN indicators showed limited incremental classification value within the present rule structure.

**Clinical trial registration:**

## 1 Introduction

Developmental dyslexia is typically characterized by persistent difficulties with accurate and/or fluent single-word reading and spelling, accompanied by disproportionate weaknesses in phonological processing and often rapid automatized naming (RAN). These cognitive markers are among the most robust correlates of word-level reading across orthographies, although individuals vary considerably in their exact profiles (1, 2). Within populations with intellectual disability (ID), similar cognitive–linguistic mechanisms appear to contribute to reading development; however, the extent to which they support valid dyslexia-like classifications remains debated. For example, phonological constraints on decoding and word recognition are frequently reported in ID, whereas RAN performance may be relatively preserved once reading level or verbal ability is controlled, suggesting that dyslexia-consistent mechanisms may occur but are not universal (3).

Whether dyslexia can be formally recognized in the context of ID depends on the diagnostic system used. In the *Diagnostic and Statistical Manual of Mental Disorders* (5th ed.; DSM-5; 4), dyslexia is specified within Specific Learning Disorder (SLD) and defined as persistent, developmentally grounded academic difficulties not better explained by intellectual disability, sensory impairment, or inadequate instruction. Although DSM-5 removed the IQ–achievement discrepancy requirement and emphasized enduring, domain-specific deficits supported by developmental and cognitive evidence, it does not permit a formal dyslexia diagnosis when criteria for intellectual disability are met; dyslexia-relevant profiles may be described functionally but are not coded as SLD (5).

By contrast, the *International Classification of Diseases* (11th ed.; ICD-11; 6) explicitly allows co-diagnosis when academic underachievement is unexpected relative to global functioning. In ICD-11, literacy difficulties are classified under Developmental Learning Disorder (DLD), while Disorders of Intellectual Development (DID) are defined primarily by limitations in intellectual and adaptive functioning, with severity indexed by everyday support needs rather than IQ bands alone. Co-diagnosis (DLD alongside DID) is permitted when academic underachievement is disproportionately severe in light of the individual’s intellectual–adaptive profile and learning opportunities, shifting the diagnostic focus from IQ discrepancy to disproportionate weakness in consolidated academic skills, such as word-level reading and spelling (7, 8). Given the current evidence indicating substantial heterogeneity and predominantly severity-based variation rather than clearly established qualitative subtypes, the study does not formulate *a priori* hypotheses. Instead, it adopts a theory-informed, question-driven approach grounded in ICD-11 diagnostic logic, allowing systematic evaluation of alternative operationalization. Within this framework, a dyslexia-consistent presentation is operationalized as enduring deficits in decoding and word recognition, supported by disproportionate weaknesses in phonological processing and, in many cases, naming-speed efficiency, documented after adequate instruction and appropriate accommodations (8, 9). Throughout, ID is used as the umbrella term, with DID and DLD reserved for explicit reference to ICD-11 nomenclature (6).

Importantly, ICD-11’s co-diagnostic logic embeds methodological guardrails that align closely with concerns in the ID literacy literature. Co-diagnosis is intended to apply only when academic deficits are disproportionate relative to both intellectual–adaptive functioning and educational exposure, reducing the risk of re-labelling commensurate delay as “specific” (7, 10–13). Measurement artefacts pose a further challenge in ID, as floor effects and construct contamination (e.g., motor, attentional, or processing-speed demands) may mimic reading-specific weakness. Consequently, best practice emphasizes multi-indicator profiling—incorporating decoding, phonological processing, RAN, and oral language—interpreted in the context of instructional history and assessment constraints (5, 14). The literature also consistently documents substantial heterogeneity in reading-related mechanisms in ID: some individuals show a clearly phonological pattern, whereas others do not, although converging evidence indicates that the same cognitive–linguistic ingredients supporting reading in the general population remain relevant in ID, albeit at lower mean levels (3, 15, 16).

Empirical research further demonstrates both continuity and divergence from typical development. Adolescents with ID often show pronounced weaknesses in decoding and word recognition relative to peers matched on verbal ability, whereas RAN and some vocabulary measures may fall within expected ranges when matched for reading level (3). Studies distinguishing lower- and higher-level comprehension processes suggest that once decoding is established, literal comprehension may be relatively adequate, while inferential and integrative processes remain vulnerable (17, 18). Early literacy research also indicates partially divergent developmental pathways in ID, with nonverbal intelligence and rhythmic timing sometimes playing a stronger scaffolding role alongside phonological awareness and letter knowledge (19, 20). At the population level, large-scale studies reveal striking heterogeneity, ranging from alphabetic and orthographic readers to individuals with minimal functional literacy, underscoring multiple developmental routes and the need for profile-sensitive classification (21). Longitudinal and quantitative syntheses similarly show that phonological awareness, RAN, and oral language predict reading accuracy and comprehension in ID in broadly similar patterns to those observed in typical development (15, 22–24).

Despite these continuities, diagnosing dyslexia within ID remains challenging. Standardized reading and phonological measures are vulnerable to floor effects and extraneous demands on attention, motor control, and processing speed, which may distort inferences about reading-specific mechanisms. The role of RAN is also contested, as its predictive contribution may reflect broader lexical retrieval and executive processes rather than a unitary causal mechanism (25). Moreover, reliance on reading-level-matched designs may inflate group differences and obscure causal interpretation (14, 26). Accordingly, whether dyslexia—defined using general-population criteria—can be meaningfully identified in mild ID depends critically on operational definitions, multi-component assessment, documentation of adequate instruction, and interpretation anchored in intellectual–adaptive functioning.

Against this background, the unresolved issue is not whether reading is delayed in ID, but whether ICD-11–guided, severity-calibrated criteria can identify a subgroup with a dyslexia-consistent profile while reducing over-identification of difficulties that may be broadly commensurate with mild ID. Building on prior multivariate work documenting substantial heterogeneity in literacy outcomes in mild nonspecific ID (16), the present study replicates the core analytic strategy in a larger sample and systematically evaluates alternative operationalization of “poor reading” and “probable dyslexia.” Specifically, we examine (a) the impact of enforcing ICD-11 co-diagnostic logic linking DLD with DID, (b) which reading components provide the most valid basis for classification, and (c) whether adding phonological-processing indicators improves the precision and specificity of subgroup identification.

Taken together, existing evidence suggests partial overlap between mechanisms underlying reading difficulties in ID and those defining dyslexia in the general population. Phonological processing and naming-speed efficiency appear central, but the boundary between achievement proportionate to global intellectual limitations and a truly disproportionate dyslexia-consistent pattern remains blurred. Consequently, classification should be grounded in converging cognitive, linguistic, and contextual indicators rather than IQ–achievement discrepancies, consistent with DSM-5’s emphasis on persistent, domain-specific difficulties and operationalized in ICD-11 through co-diagnosis when unexpected underachievement is demonstrated (5, 27, 28). Within this framework, we address four research questions: (RQ1) Can ICD-11 diagnostic criteria for developmental dyslexia be validly applied in individuals with mild ID? (RQ2) Does enforcing ICD-11 co-diagnostic logic improve the specificity and construct validity of dyslexia-like classifications in mild ID? (RQ3) Which reading components most validly support classification as “poor readers,” and does reading comprehension show incremental validity beyond word-level reading? (RQ4) Does incorporating phonological-processing measures improve the precision and specificity of identifying a “probable dyslexia” subgroup beyond word-level reading alone? Each research question reflects a specific theoretical or diagnostic issue: the validity of applying ICD-11 criteria (RQ1), the role of severity anchoring in defining unexpected underachievement (RQ2), the centrality of word-level reading components (RQ3), and the incremental value of phonological and naming-speed indicators (RQ4).

## 2 Materials and methods

### 2.1 Participants

The study sample was drawn from a nationwide database comprising diagnostic records of children and adolescents assessed using the Specialist Battery for the Assessment of Cognitive Abilities and Academic Skills (SB6/18, see Appendix 1). The database integrated data from three sources: (a) a validation study of the SB6/18 (29), (b) assessments of students with mild intellectual disability conducted in mainstream and special schools (16), and (c) the Diagnostic Support System (DSS) database, which aggregates SB6/18 assessments collected in psychological and educational counselling centers and schools across Poland. The combined data pool consisted of 12,390 individual records (see Data Availability Statement). In the Polish educational system, assessment in psychological and educational counselling centers is broadly accessible and often required to formalize adapted educational provision for students with mild ID. Thus, the present sample reflects a broad service-based diagnostic cohort rather than a tertiary-clinic sample, although it is not population-based and may still over-represent students with more marked difficulties.

From this pool, 446 observations were selected for the present analyses based on the following inclusion criteria: (a) availability of complete scores on six indicators central to the research questions—Phonetic Coding–Linguistic Aspect (PC-LA), Phonetic Coding–Cognitive Complexity (PC-CC), Rapid Automatized Naming (RAN), Decoding (D), Reading Fluency (RF), and Reading Comprehension (RC); (b) complete data for core demographic variables (age, gender, and place of residence); and (c) formal confirmation of a diagnosis of mild intellectual disability (mild ID) issued within the official diagnostic system. These criteria were designed to ensure comparability across participants and to focus the analysis on core reading and cognitive–linguistic indicators relevant to the research questions, while maintaining a sufficiently large and ecologically valid clinical sample.

Information on parental educational attainment (mother and father separately) was included to provide a broader sociodemographic context. However, because these variables were not mandatory components of the diagnostic procedure, they contained substantial missing data and were therefore treated descriptively only. **Table 1** presents the detailed composition of the study sample.

**Table 1:** Sociodemographic characteristics and data sources of the study sample of students with mild intellectual disability (*N* = 446).

Variable	N	%	Age			
			Min	Max	M	SD
Total	446	100	6:5	18:8	12.56	2.70
Gender						
female	184	41	6:8	18:4	12.71	2.65
male	262	59	6:5	18:8	12.46	2.73
Residence						
city	291	65	6:8	18:8	12.62	2.63
countryside	155	35	6:5	18:4	12.45	2.83
Mother’s education						
primary or lower secondary	40	9	6:8	16:1	12.04	2.45
secondary	45	10	7:3	17:4	12.71	2.45
vocational	81	18	6:5	18:8	12.92	2.47
higher	19	4	7:3	15:5	12.12	2.32
missing data	261	59	6:8	18:7	12.54	2.87
Father’s education						
primary or lower secondary	40	9	6:8	18:8	12.18	2.62
secondary	27	6	7:3	16:8	12.45	2.34
vocational	90	20	6:5	18:4	13.05	2.29
higher	16	4	7:3	15:5	11.42	2.46
missing data	273	61	6:8	18:7	12.54	2.86
Source of data						
Diagnostic system	336	75	6:5	18:7	12.26	2.92
Schools	91	20	9:11	18:8	13.43	1.58
Validation study	19	5	11:11	17:2	13.70	1.53

Age is reported in years: months for minimum and maximum values; for the calculation of means and standard deviations, months were converted into fractions of a year (denominator = 12).

The sample covered a broad developmental age range, spanning from early primary school to late adolescence, with a mean age in early adolescence. This wide age distribution reflects the real-world structure of the diagnostic system from which the data were drawn and aligns with the study’s aim of examining heterogeneity in reading-related profiles across developmental stages within mild intellectual disability. Gender distribution was moderately imbalanced, with boys more frequently represented than girls, a pattern commonly observed in diagnoses of intellectual disability and learning difficulties (30, 31). Participants were drawn from both urban and rural areas, with a predominance of city residents, supporting the ecological validity of the findings across educational contexts. However, the available parental education data indicated substantial socio-educational diversity, although the high proportion of missing values limited firm conclusions.

Most observations originated from the national diagnostic system, supplemented by smaller subsamples from school-based assessments and the SB6/18 validation study. These data sources differed slightly in age composition, reflecting variation in assessment contexts and referral pathways. Nevertheless, all participants met identical inclusion criteria for cognitive–linguistic and reading measures, supporting the integration of data across sources for the present analyses.

Overall, the final sample represents a clinically and educationally realistic cohort of students with mild intellectual disability, characterized by substantial heterogeneity in age, background, and assessment context. This heterogeneity is central to the study’s objectives, as it enables a robust examination of variability in reading components and cognitive–linguistic profiles while requiring analytic strategies that are sensitive to developmental and contextual influences rather than reliance on narrow or artificially homogeneous subsamples.

### 2.2 Procedure and measures

All measures were obtained using the SB6/18 test battery (see Appendix 1), a standardized instrument designed to assess cognitive abilities and school-related skills in children and adolescents (29, 32).

All subtest outcomes were converted to sten scores (*M* = 5.5, *SD* = 2) using normative tables, with scores of 1–3 indicating low performance. In the present study, sten = 1 was treated as an indicator of extremely low performance on age-normed metrics and used as a pragmatic severity anchor within mild ID; it was not intended as a direct operationalization of unexpected underachievement relative to intellectual and adaptive functioning in the strict ICD-11 sense. SB6/18 stens are based on age-smoothed norming procedures that correct for skewness in raw-score distributions (29). Consequently, analyses based on sten scores attenuate age-related performance differences that would otherwise be evident in raw scores, supporting comparability across participants of different ages.

The SB6/18 battery demonstrates strong psychometric properties, including high internal consistency across subtests (α = 0.74–0.98), excellent reliability for the global index (α = 0.91), and high test–retest stability (r = 0.73–0.94; 29). Confirmatory factor analyses support the theoretical structure of the battery, consistent with the Cattell–Horn–Carroll framework, with good model fit (RMSEA = 0.073; CFI = 0.94).

Data were collected individually by trained professionals under standardized testing conditions in accordance with SB6/18 administration guidelines. Written informed consent was obtained from participants’ legal guardians. The study was approved by the Ethics Committee for Research Projects at the Faculty of Social Sciences, University of Gdansk, Poland (decision no. 13/2022) and complied with the Declaration of Helsinki.

### 2.3 Statistical analysis

To address the research questions, the analysis combined a person-centered profiling approach with a condition-based, criterion-referenced analysis using ICD-11-guided, severity-calibrated classification rules. All analyses were conducted on standardized scores (sten scores) derived from the SB6/18 battery to ensure comparability across age and assessment contexts.

#### 2.3.1 Preliminary analyses

Descriptive statistics (means, standard deviations, skewness, and kurtosis) were computed for the six key variables: D, RF, RC, PC–LA, PC–CC, and RAN. Pearson correlation coefficients were calculated to examine the strength and coherence of associations among word-level reading, reading comprehension, and cognitive–linguistic measures. This served two purposes: (a) verify that the interrelations among variables were consistent with established models of reading development and dyslexia, and (b) inform the multivariate analyses by documenting shared and distinct variance among reading and phonological components.

#### 2.3.2 Part 1: cluster analysis of reading and cognitive–linguistic profiles

The first analytic component consisted of a replication and extension of a previously published cluster analysis (16), conducted on a substantially larger and more heterogeneous sample, while employing a modified set of indicators explicitly selected to reflect the core components of developmental dyslexia as defined in ICD-11. The primary goal was to examine whether within the population of students with mild ID naturally emerging multivariate profiles could be identified that were characterized by reading-specific difficulties consistent with the ICD-11 diagnostic criteria for developmental dyslexia, thereby providing an empirical test of the applicability of these criteria in individuals with mild ID (RQ1).

Hierarchical cluster analysis was performed using Manhattan distance and complete linkage (33). Manhattan distance is less sensitive to extreme values than Euclidean distance and is well-suited for capturing profile-based differences across multiple standardized indicators. Second, complete linkage tends to produce relatively compact and well-separated clusters and reduces chaining effects, which is desirable given the substantial heterogeneity of reading-related cognitive profiles in individuals with mild ID. Third, hierarchical clustering allows for a systematic evaluation of alternative cluster solutions without imposing an *a priori* number of clusters. To reduce the influence of floor effects, analyses were based on multiple indicators and robust distance metrics (Manhattan distance), and cluster stability was evaluated using bootstrap resampling rather than relying on a single partition.

To determine the optimal number of clusters, a bootstrap resampling procedure with Jaccard similarity coefficients was applied (34). A total of 2,000 bootstrap replications were conducted for solutions ranging from 2 to 10 clusters. For each solution, the stability of individual clusters was quantified using the Jaccard coefficient, with values ≥.75 indicating high stability. Consistent with prior recommendations, values below .60 were treated as indicating substantial instability. In selecting the solution for interpretation, we required that no cluster in a given solution fall below .60 (to avoid highly unstable partitions), while additionally reporting the number of highly stable clusters (Jaccard ≥.75) for each candidate solution.

To characterize the clusters, between-cluster differences were tested using independent-samples Welch’s *t*-tests for each of the six variables, with Cohen’s *d*. These comparisons were not intended as confirmatory hypothesis tests, but as descriptive tools to quantify the magnitude and pattern of differences between empirically derived profiles. The resulting effect sizes provided information on which reading and cognitive–linguistic components most strongly differentiated subgroups, directly informing RQ3 and setting the stage for the diagnostic-criterion analyses.

#### 2.3.3 Part 2: frequency analysis of dyslexia-relevant diagnostic conditions

The second analytic component examined the validity and specificity of dyslexia-like classifications in mild ID by analyzing the frequency and distribution of theoretically motivated diagnostic conditions (RQ1, RQ2 and RQ4). Dyslexia-consistent profiles were operationalized using explicit logical conditions guided by ICD-11 co-diagnostic logic and core definitional features of dyslexia, rather than cluster membership alone.

Four binary conditions were defined using standard sten cut-offs for low (sten 1–3) and very low (sten 1) performance: (A1) low word-level reading or reading comprehension (D or RF, or RC ≤ 3 sten); (A_2_) very low word-level reading or reading comprehension (D or RF, or RC = 1 sten); (A_3_) very low decoding specifically (D = 1 sten); (B) low performance on at least one phonetic coding or RAN measure (PC–LA or PC–CC or RAN ≤ 3 sten).

These conditions were combined into six diagnostic groupings reflecting alternative operationalization of dyslexia: (G1) broad ICD-11 literacy impairment criteria (A1 and B); (G2) ICD-11-aligned criteria with an ID-specific severity requirement (A2 and B); (G3) probable dyslexia within mild ID based on severe decoding impairment (A3 and B); and residual groups (G4–G6) capturing profiles without phonological deficits or with severe reading impairment alone. The decoding-based definition (G3) was treated as the closest ICD-11-guided proxy for a dyslexia-consistent presentation within mild ID, while broader definitions (G1 and G2) were used to evaluate potential over-identification.

For each grouping, absolute and relative frequencies were calculated and cross-tabulated with cluster membership. This approach allowed assessment of whether increasingly stringent, ID-sensitive criteria reduced over-identification and yielded classifications aligned with empirically derived profiles (RQ2). Comparisons across groupings further tested whether enforcing ICD-11 co-diagnostic logic improved specificity relative to general-population dyslexia definitions.

Finally, overlap between phonological-processing conditions (B) and severe decoding impairment (A3) was examined to address RQ4, evaluating whether inclusion of phonological indicators meaningfully refined identification of a probable dyslexia subgroup beyond word-level reading measures alone.

#### 2.3.4 Analytic rationale and integration

The two analytic components served complementary purposes. The cluster analysis addressed whether dyslexia-consistent profiles emerge naturally at the multivariate level in mild ID, supporting or challenging the validity of applying standard dyslexia constructs (RQ1). The condition-based frequency analyses translated these profiles into diagnostically interpretable classifications, allowing explicit evaluation of ICD-11 logic, severity thresholds, and the incremental value of phonological processing measures (RQ2–RQ4). This integrated strategy explicitly combines an exploratory component (cluster analysis) with a theory-driven, condition-based evaluation of ICD-11 diagnostic logic. The aim is not to test narrowly specified hypotheses, but to assess the behavior of theoretically grounded classification rules under varying severity constraints.

## 3 Results

### 3.1 Descriptive statistics and associations between reading and cognitive–linguistic measures

**Table 2** presents descriptive statistics and intercorrelations for the six standardized (sten) indicators included in the analyses. Overall, substantial variability was observed in reading and cognitive–linguistic performance among students with mild ID. Word-level reading measures (D and RF) showed low mean scores and moderate positive skewness, indicating clustering at the lower end of the performance range. RC exhibited the lowest mean and the greatest deviation from normality, consistent with pronounced difficulties in higher-level text processing.

**Table 2:** Descriptive statistics and Pearson correlations among standardized reading and cognitive–linguistic measures (SB6/18).

Variable	M	SD	Skew.	Kurt.	Correlation				
					RF	RC	PC–LA	PC–CC	RAN
Decoding (D)	3.00	2.11	0.82	-0.32	.70	.54	.44	.31	.24
Reading Fluency (RF)	2.40	1.52	0.91	-0.02		.61	.45	.28	.19
Reading Comprehension (RC)	1.75	1.25	1.79	2.67			.40	.23	.22
Phonetic Coding – Linguistic Aspect (PC–LA)	2.12	1.39	1.07	0.54				.46	.24
Phonetic Coding – Cognitive Complexity (PC–CC)	2.74	1.22	0.78	1.04					.12
Rapid Automatized Naming (RAN)	3.17	1.74	0.53	-0.44					

All correlations are significant with p <.05.

Correlational analyses revealed a coherent and theoretically interpretable pattern. D and RF were strongly associated, indicating that word-level processes form a tightly coupled core of reading performance. RC showed moderate associations with both decoding and fluency, suggesting partial dependence on word-level skills alongside additional linguistic and cognitive demands. PC–LA was moderately correlated with all reading measures, whereas PC–CC showed weaker but significant associations. RAN was modestly related to word-level reading and comprehension, supporting its role as a complementary efficiency-related component rather than a primary determinant of reading accuracy. This pattern is consistent with established models of dyslexia and provided an empirical basis for subsequent multivariate and criterion-based analyses (RQ3, RQ4).

### 3.2 Cluster analysis of reading and cognitive–linguistic profiles (RQ1, RQ3)

Bootstrap stability analyses using Jaccard similarity coefficients (**Table 3**) indicated that the two-cluster solution was the only partition in which no cluster fell below the .60 stability threshold. Solutions with a greater number of clusters increasingly produced at least one unstable cluster and were therefore not retained.

**Table 3:** Bootstrap stability of hierarchical cluster solutions (2–10 clusters) based on Jaccard similarity coefficients.

Number of clusters tested	Stable clusters (n)	Jaccard similarity coefficient			
		Mean	Median	Min	Max
2	1	0.82	0.82	0.68	0.96
3	2	0.70	0.76	0.46	0.88
4	2	0.61	0.62	0.40	0.80
5	1	0.55	0.51	0.31	0.75
6	1	0.55	0.48	0.38	0.83
7	1	0.50	0.48	0.24	0.82
8	1	0.50	0.48	0.26	0.80
9	1	0.48	0.47	0.28	0.78
10	1	0.47	0.43	0.30	0.76

Stable clusters are defined as clusters with a Jaccard similarity coefficient ≥.75; values <.60 indicate substantial instability.

**Figure 1:**
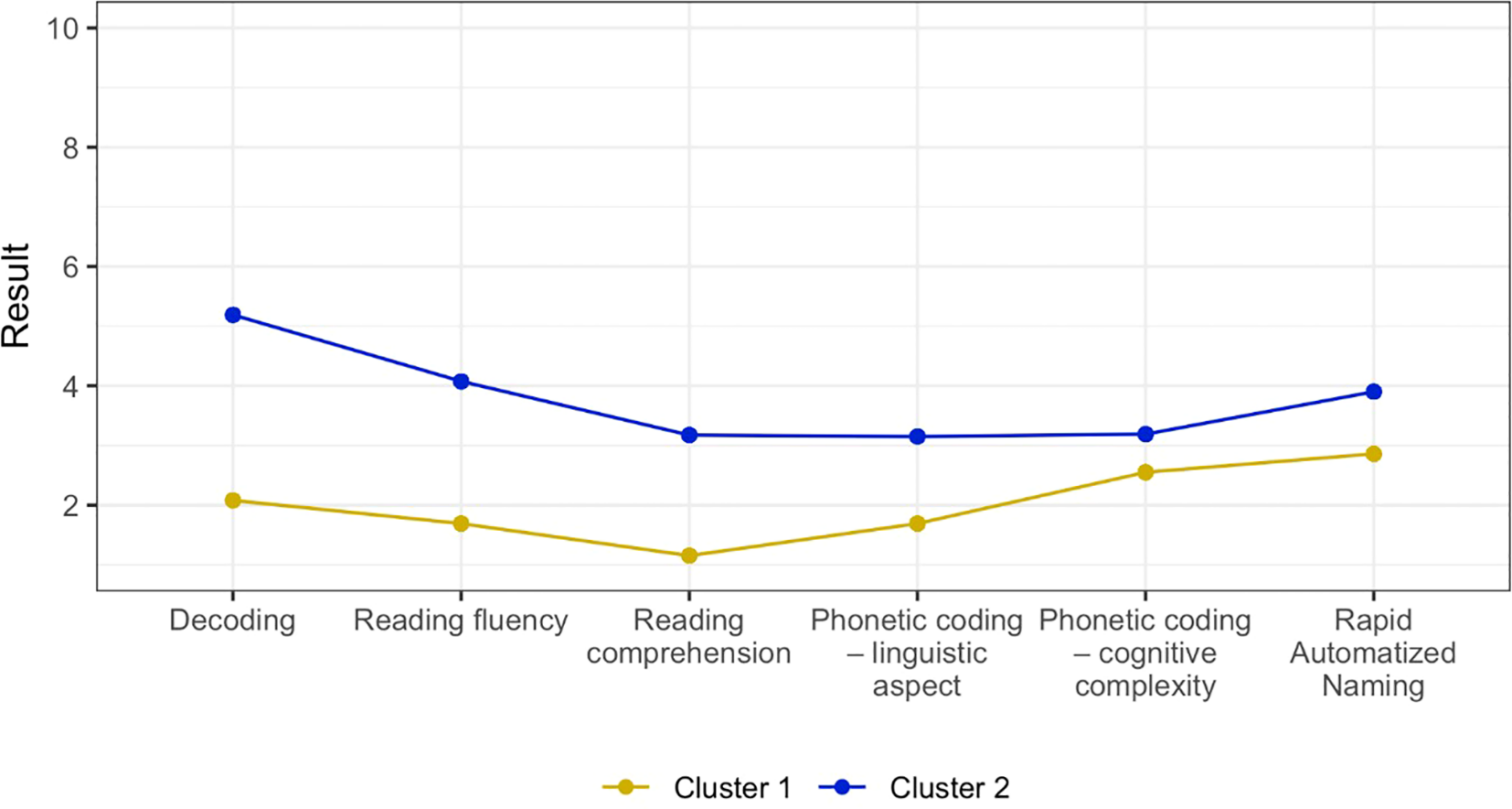
Mean standardized (Sten) scores for reading and cognitive–linguistic measures across the two empirically derived clusters.

The two clusters differed markedly across all six indicators (**Figure 1**; **Table 4**). Cluster 1, comprising the majority of participants, was characterized by uniformly low performance across decoding, reading fluency, reading comprehension and phonological-processing measures. Cluster 2 showed higher scores across all domains, although performance remained below population norms. Between-cluster comparisons yielded large effect sizes for D, RF, RC and PC–LA, and moderate effect sizes for PC–CC and RAN. This pattern indicates that profiles were primarily differentiated by severity and pervasiveness of reading-related difficulties rather than by qualitatively distinct mechanisms.

**Table 4:** Between-cluster comparisons of reading and cognitive–linguistic measures: means, t-tests, and effect sizes.

Variable	Cluster 1				Cluster 2				Welch’s t-test					Cohen’s d	
	N	M	SD		N	M	SD		Est.	df	p	Est.		Magnitude
D	314	2.08	1.47		132	5.19	1.77		-17.74	210.29	<.001	-1.91		large
RF	314	1.69	0.98		132	4.08	1.23		-19.84	204.13	<.001	-2.15		large
RC	314	1.15	0.43		132	3.17	1.40		-16.27	141.27	<.001	-1.95		large
PC–LA	314	1.69	1.10		132	3.15	1.46		-10.30	195.95	<.001	-1.13		large
PC–CC	314	2.55	1.08		132	3.19	1.40		-4.68	198.85	<.001	-0.51		moderate
RAN	314	2.86	1.58		132	3.90	1.89		-5.57	212.49	<.001	-0.60		moderate

D, Decoding; RF, Reading Fluency; RC, Reading Comprehension; PC–LA, Phonetic Coding – Linguistic Aspect; PC–CC, Phonetic Coding – Cognitive Complexity; RAN, Rapid Automatized Naming.

The cluster structure closely mirrored that reported in the earlier, smaller-scale study, despite differences in sample composition and indicator selection. This replication supports the robustness of the identified profiles and suggests that reading difficulties in mild ID organize along a stable severity dimension (RQ1). At the same time, the absence of a distinct phonological-only or naming-speed-only cluster highlights heterogeneity in underlying mechanisms and cautions against simple categorical interpretations (RQ3). This interpretation was further supported by sensitivity analyses using alternative clustering specifications (Euclidean distance and Ward’s method), which yielded a comparable two-cluster solution and a highly similar pattern of severity-based differentiation across indicators, confirming the robustness of the identified structure (see *Supplementary Materials*).

### 3.3 Frequency analysis of dyslexia-relevant diagnostic conditions (RQ1, RQ2, RQ4)

**Figure 2:**
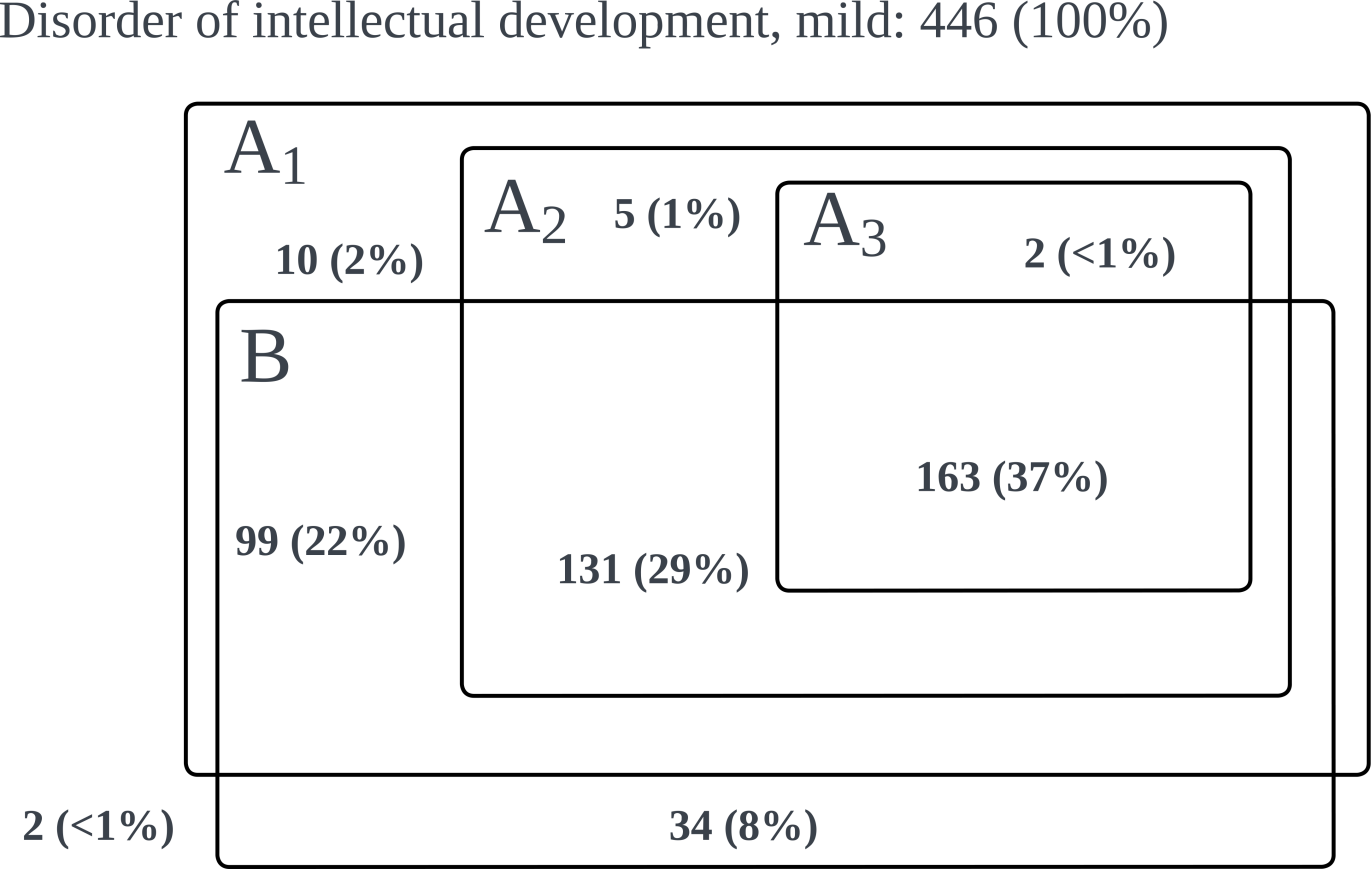
Prevalence of dyslexia-relevant diagnostic conditions (A_1_–B) in students with mild intellectual disability.

**Figure 2** summarizes the prevalence of individual diagnostic conditions (A_1_–B) in the full sample, showing that low reading performance and phonological-processing weaknesses were both highly prevalent, but not perfectly overlapping. **Table 5** presents the distribution of participants across diagnostic groupings and their correspondence with cluster membership.

**Table 5:** Frequencies of ICD-11–based dyslexia classification groups and their distribution across empirically derived clusters.

Group		Condition	In the full sample		N (%) in clusters	
			N	%	Cluster 1	Cluster 2
G1	The general ICD-11 dyslexia criteria	A_1_ and B	393	88	308 (98)	85 (64)
G2	ICD-11 dyslexia criteria with an ID-specific additional requirement	A_2_ and B	294	66	279 (89)	15 (11)
G3	ICD-11 dyslexia criteria with an ID-specific additional requirement - adjusted	A_3_ and B	163	37	160 (51)	3 (2)
G4	No phonological difficulties	not B	19	4	6 (2)	13 (10)
G5	Very low reading (decoding) level without phonological difficulties	A_3_ and not B	2	<1	2 (1)	–
G6	Very low reading (decoding) level	A_3_	165	37	162 (52)	3 (2)

Applying a general ICD-11 dyslexia definition (G1: A1 and B) yielded a very high classification rate, encompassing most of the sample, including nearly all individuals in Cluster 1 and a substantial proportion of Cluster 2. This pattern indicates low specificity when general-population criteria are applied without adjustment for intellectual disability, supporting concerns about over-identification (RQ1).

Introducing a more stringent severity requirement (G2: A2 and B) substantially reduced classification prevalence and concentrated classified cases within Cluster 1 while largely excluding Cluster 2. The most restrictive operationalization (G3: A3 and B), emphasizing very low D in combination with phonological weaknesses, produced the smallest and most homogeneous subgroup, almost entirely confined to Cluster 1. These proportions should not be interpreted as population prevalence estimates, as the sample was drawn primarily from a diagnostic support system and may over-represent students with more severe educational difficulties. The reliance on a clinically and diagnostically referred sample further limits generalizability and may introduce sampling bias, including potential inflation of classification rates. As such, the reported frequencies should not be interpreted as population prevalence estimates. This supports the use of an extreme-severity cutoff as a pragmatic marker of very severe reading difficulty in mild ID. However, this criterion alone does not establish that reading performance is unexpectedly low relative to overall functioning. Accordingly, this subgroup may be interpreted cautiously as representing a “probable dyslexia” profile within mild ID. This pattern suggests greater classificatory selectivity, but it does not by itself establish diagnostic validity.

Residual groups without phonological difficulties (G4–G6) were rare and primarily associated with Cluster 2, suggesting that severe decoding impairment in the absence of phonological weaknesses is uncommon in this population. Comparison of G6 (very low decoding alone) and G3 (very low decoding plus phonological or RAN deficits), conducted to address RQ4, showed nearly identical group sizes and highly similar cluster distributions. This indicates little incremental classification gain from adding phonological or RAN criteria once an extreme decoding threshold is applied, likely reflecting substantial shared variance between severe decoding impairment and phonological processing weaknesses. However, this finding should be interpreted cautiously, because decoding is downstream of phonological processing and naming efficiency; thus, requiring very low decoding may partly preselect individuals who already show weaknesses in these related domains. Thus, phonological and RAN indicators may contribute more to clinical formulation than to further narrowing classification under these conditions.

## 4 Discussion

This study examined whether a dyslexia-consistent subgroup can be meaningfully identified within mild ID when ICD-11-guided, severity-calibrated rules are applied, and whether phonological processing measures and RAN improve subgroup specificity beyond word-level reading indicators. Across analyses, two overarching patterns emerged. First, reading and related cognitive–linguistic performance was best characterized by a severity gradient rather than by clearly separable, qualitatively distinct profiles. Second, when dyslexia criteria are applied without an ID-sensitive expectation anchor, subgroup assignment becomes highly inclusive and therefore poorly specific, particularly for students whose reading performance is broadly consistent with their general cognitive level.

### 4.1 Reading profiles within mild ID: severity over qualitative subtypes

The cluster solution identified two robust groups that differed markedly in decoding, reading fluency, and reading comprehension, with smaller differences in phonetic coding (linguistic and cognitive-complexity aspects) and RAN. The coherence and magnitude of these differences suggest that, in mild ID, reading difficulties tend to co-occur across components, with word-level reading marking the steepest separation. This pattern aligns with prior evidence indicating substantial heterogeneity in literacy within mild ID and borderline intellectual functioning, where group differences more often reflect degree of difficulty than a distinct dyslexia-like cognitive signature (21, 26). Narrative syntheses similarly note that although phonological and fluency weaknesses are common, the field has not converged on stable, mechanism-based subtypes comparable to those proposed in typical development dyslexia research (14, 35). Accordingly, the identified groups are best interpreted as severity-based profiles rather than qualitatively distinct dyslexia-like subtypes, consistent with person-centered approaches in which profiles may differ in overall level of performance rather than configuration of indicators.

Correlational findings further support a severity-based interpretation. Decoding was strongly associated with reading fluency (r = .70) and moderately with reading comprehension (r = .54), whereas associations between word-level reading and phonetic coding were moderate (e.g., decoding with phonetic coding–linguistic aspect r = .44). In contrast, correlations with RAN were small (e.g., decoding r = .24; reading fluency r = .19). This pattern is consistent with the view that, in mild ID, individual differences in word-level reading are central, while reading comprehension is additionally constrained by broader language and cognitive factors and is vulnerable to measurement floor effects (17, 18, 22, 23).

Importantly, the reading comprehension measure showed substantial floor effects and positive skew, which may have constrained its ability to differentiate among lower-performing individuals and reduced its contribution to distinguishing profiles. While the overall pattern of results was supported by multiple indicators and robust cluster stability, suggesting that the findings were not solely driven by restricted variability at the lower end of the scale, this limitation is particularly relevant for clustering analyses, as variables with restricted variance contribute less to distance-based solutions. Consequently, the relatively stronger role of word-level measures in distinguishing profiles should be interpreted with caution, as it may partly reflect measurement limitations rather than purely substantive differences in the relative importance of reading components.

### 4.2 Implications for ICD-11 and DSM logic: approximating “Unexpectedness” in ID

A key contribution of this study is to show that classification rates depend strongly on how severity thresholds are specified in the context of mild ID. Importantly, our analyses do not provide a strict test of ICD-11 unexpected underachievement, because reading performance was not directly compared with each participant’s intellectual and adaptive functioning. Rather, they examine whether increasingly stringent, ID-sensitive severity anchors reduce over-identification. When a general ICD-11 dyslexia definition was applied without an ID-sensitive severity anchor (Group G1: A1 + B), 88% of the sample met criteria, including 64% of the higher-performing cluster. Such prevalence is difficult to reconcile with dyslexia as a specific learning disorder characterized by persistent reading difficulties not fully explained by global developmental level. DSM-5 addresses this concern by requiring learning difficulties to be “in excess of those usually associated” with intellectual disability (4), and contemporary frameworks similarly emphasize evaluating performance relative to overall cognitive and adaptive functioning (5).

Introducing an ID-referenced severity requirement was associated with greater classificatory specificity. Applying a more stringent criterion (Group G2: A2 + B) reduced classification to 66% overall and concentrated cases in the more impaired cluster (89% in Cluster 1 vs. 11% in Cluster 2). Further tightening severity (Group G3: A3 + B) reduced classification to 37% and almost exclusively identified students in the lower-performing cluster. The relatively high prevalence even under this restrictive definition may reflect the use of diagnostic support system data, which likely over-represent students referred for educational and psychological assistance and, therefore, those with more severe difficulties than would be expected in the broader mild ID population. Estimating the population-level prevalence of a “probable dyslexia” profile among individuals with ID will require large-scale, representative epidemiological studies. These findings suggest that explicit, ID-sensitive severity anchors may improve specificity in ICD-11-guided research on dyslexia in mild ID. However, such anchors should be interpreted as pragmatic proxies and not as a direct demonstration of disproportionate academic underachievement relative to global functioning. More broadly, the present findings are relevant to ongoing discussions on the definition of dyslexia (36), particularly regarding the role of severity, heterogeneity, and the operationalization of core diagnostic criteria. In line with recent work revisiting dyslexia definitions, the results highlight the importance of explicitly specifying thresholds and decision rules, especially in populations where low achievement is expected (5).

### 4.3 Reading components supporting subgroup identification

Across analyses, word-level measures, particularly decoding and reading fluency, were the most discriminative indicators. This finding is consistent with evidence that students with ID often exhibit pronounced difficulties with fluent word identification, even when grapheme–phoneme conversion is relatively preserved in transparent orthographies (26). It also aligns with Simple View–based findings in mild ID showing that decoding contributes substantially to reading comprehension but does not fully account for it, with linguistic comprehension and broader language skills explaining additional variance (17, 18, 23). More broadly, this pattern is consistent with componential models of reading in which word-level decoding constrains comprehension but does not exhaust the contribution of language-related processes (37, 38). In the present dataset, reading comprehension exhibited the lowest mean and greatest skew, increasing the likelihood of floor effects and limiting its utility for differential classification when the goal is to identify dyslexia-consistent subgroups rather than overall academic functioning.

Finally, the close correspondence between the cluster structure observed here and solutions derived using alternative indicator sets reinforces the conclusion that, in mild ID, a single dominant dimension of reading severity may overshadow finer-grained cognitive dissociations. This does not imply that component-level mechanisms are unimportant, but rather that common co-occurrence and measurement constraints may limit the incremental classification value of additional indicators in cross-sectional analyses.

### 4.4 Phonological processing and RAN: theoretically relevant but limited incremental classification value

Phonological weaknesses are widely implicated in dyslexia and are also frequently observed in ID (2, 3, 35). In typical development, RAN is robustly associated with reading fluency and can explain variance beyond phonological skills, particularly in transparent orthographies where accuracy reaches ceiling earlier and speed becomes the primary marker (39–41). Against this backdrop, phonetic coding and RAN differences between clusters were present but smaller than word-level reading differences, and incorporating phonological difficulty criteria resulted in only modest changes in subgroup allocation once severe decoding impairment was required. The limited incremental contribution of phonological and RAN measures may reflect both substantive and methodological factors. On the one hand, severe decoding impairment may already capture variance associated with these processes in mild ID. On the other, this pattern may be influenced by restricted variability, floor effects, dichotomization, and the limited operationalization of phonological processing. Accordingly, this finding should be interpreted as limited incremental classification value within the present framework rather than evidence against the importance of these constructs.

Two conservative interpretations may account for this pattern without discounting phonological or naming-speed mechanisms. First, decoding is downstream of phonological and automaticity-related processes; when classification already requires very low decoding, adding a correlated phonological criterion may yield limited incremental separation because many individuals meeting the decoding threshold also show phonological weaknesses. Second, the operationalization of phonological difficulty is critical. Phonetic coding measures may capture only selected phonological processes, and dichotomizing performance into difficulty present/absent can reduce sensitivity to graded variation. In transparent orthographies, developmental shifts from accuracy to speed may further elevate the relative contribution of naming-speed/automaticity measures for fluency, while phonological measures remain more predictive earlier in development (41, 42). These findings should also be interpreted in light of the Polish orthographic context. Polish is a relatively transparent orthography for reading (often described as semi-transparent overall; 43), so the relative contribution of phonological and naming-speed measures may differ from that observed in more opaque writing systems. Together, these considerations indicate that phonological processing and RAN remain theoretically important, but their incremental diagnostic value depends on how dyslexia is operationalized in ID and on the measurement model employed.

### 4.5 Clinical and educational implications

From a clinical perspective, the findings support a two-step approach to classification: first, quantify reading impairment with emphasis on word-level decoding and fluency; second, interpret severity relative to global intellectual–adaptive functioning to determine whether difficulties plausibly reflect a specific disorder rather than a general developmental constraint (4–6). This supports the use of ID-sensitive decision rules that preserve the core dyslexia construct while limiting over-identification.

Educational implications are similarly pragmatic. Systematic reviews indicate that students with ID benefit from structured reading interventions incorporating phonological awareness, letter–sound instruction and decoding practice, yielding small-to-moderate improvements in foundational reading outcomes (44). Meta-analytic evidence also supports phonics-focused decoding instruction for learners with ID, with large effects reported across controlled and single-case designs, although the broader evidence base remains limited and heterogeneous (45, 46). Accordingly, irrespective of diagnostic classification, the high prevalence of low word-level reading supports access to explicit, systematic instruction rather than treating low achievement as an immutable correlate of ID.

### 4.6 Limitations and directions for future research

Several limitations temper interpretation. First, the cross-sectional design precludes conclusions about developmental change; longitudinal studies are needed to examine whether ID-sensitive dyslexia classifications predict differential trajectories and response to intervention, which would strengthen construct validity. Accordingly, the present classification results should be interpreted as provisional rather than diagnostically definitive. The wide age range of the sample introduces additional heterogeneity, particularly for reading skills such as decoding and fluency, which are sensitive to cumulative instructional exposure and educational attainment. Although age-standardized scores were used to improve comparability across participants, residual developmental and experiential effects cannot be fully ruled out. Second, the reading comprehension measure exhibited substantial floor effects and skew, potentially limiting its discriminative value and obscuring heterogeneity at higher levels of text processing. Third, phonological ability was indexed using phonetic coding measures; future research should employ a broader battery (e.g. phoneme awareness, nonword repetition, phonological memory) and avoid overly coarse dichotomization. This finding may also reflect methodological constraints (e.g., restricted variability and limited operationalization), and should therefore be interpreted cautiously. The absence of formally specified hypotheses reflects the current state of theory in this area and the study’s partly exploratory design; future research may build on these findings to develop more precise, testable models.

Although the findings align with the intent of ICD-11 co-diagnosis guidelines to reserve additional learning disorder specifiers for atypically severe learning difficulties in the context of DID, unexpectedly poor reading in the present study was operationalized as sten 1 (very low performance relative to population norms), rather than as unexpected underachievement in the strict ICD-11 sense. Finally, the sample was restricted to mild ID within a specific educational and orthographic context; generalization to other severity levels, languages and instructional contexts requires replication.

An additional limitation is the lack of detailed diagnostic information beyond the formal classification of mild intellectual disability. The available dataset did not include fine-grained measures of intellectual and adaptive functioning, which limits the ability to contextualize reading performance relative to individual profiles and may constrain internal validity.

## 5 Conclusion

The findings indicate that dyslexia-consistent reading profiles can be identified in mild ID, but classification prevalence and subgroup coherence are highly sensitive to how severity thresholds are operationalized. Broad general-population criteria proved over-inclusive, whereas more stringent cut-offs concentrated cases within the lower-performing profile. Word-level reading—particularly decoding and reading fluency—provided the most robust basis for subgroup identification, while phonological processing and RAN, although theoretically relevant, showed limited incremental classification value within the present rule structure, likely in part because severe decoding impairment already captures downstream consequences of weaknesses in related domains. Adopting ID-sensitive, severity-calibrated decision rules is therefore essential to reduce over-inclusiveness and to preserve scientific coherence and clinical utility when applying dyslexia constructs in mild ID.

## Data Availability

The datasets presented in this study can be found in online repositories. The names of the repository/repositories and accession number(s) can be found below: The data and R code required to reproduce the statistical analyses reported in this article are openly available in the Open Science Framework (OSF) repository at: https://osf.io/5p4bg/overview?view_only=861d599b2d79495092fc62ae07900a0a.
