# Supplementary material for: Identifying dyslexia-consistent reading profiles in mild intellectual disability: cluster-derived severity gradients and severity-calibrated classification rules
Source: Front Psychiatry. 2026 May 29;17:1805069. doi: 10.3389/fpsyt.2026.1805069 (PMC13260349; doi:10.3389/fpsyt.2026.1805069)
Supplement: Supplementary file 1 [file Table1.docx]

Appendix 1

**Structure of the *Specialist Battery for the Assessment of Cognitive Abilities and Academic Skills (SB6/18)***

The Specialist Battery for the Diagnosis of Cognitive Abilities and School Skills (SB6/18) is a standardized psychometric instrument designed to evaluate a broad spectrum of cognitive processes and academic competencies that are critical for learning. Its architecture is grounded in the Cattell–Horn–Carroll (CHC) theory of cognitive abilities, thereby incorporating both broad ability domains and more circumscribed, narrow constructs.

The battery comprises six core scales, each indexing a distinct functional domain: visuospatial processing, auditory–linguistic processing, processing speed, long-term memory, knowledge, and reading and writing. Each scale is divided into subscales that correspond to specific processes or skill sets, which are evaluated through one or more psychometric tests or indicators.

***The most important psychometric properties of the SB6/18***

The psychometric analysis of the SB6/18 battery demonstrates excellent measurement properties across its scales, subscales, and individual tests. Internal consistency, as assessed by Cronbach’s alpha, ranged from **α = 0.74 to α = 0.98** across subtests, indicating high reliability for both cognitive and academic skill measures. Additionally, the overall reliability coefficient for the cognitive and academic skills quotient was remarkably high (**α = 0.91**), suggesting that the battery provides a stable and consistent evaluation of learning-relevant cognitive processes.

Test-retest reliability estimates for a selected group of participants ranged from **r = 0.73 to 0.94**, supporting the temporal stability of SB6/18 outcomes. The confirmatory factor analysis (CFA) provided strong evidence of the battery’s structural validity, with fit indices indicating an excellent model fit **(root mean square error of approximation,** **RMSEA = 0.073, comparative fit index, CFI = 0.94**). These results confirm that the assumed hierarchical structure—aligned with the CHC theory of cognitive abilities—is well represented in the data.

Evidence for convergent validity was observed through strong correlations with other established measures of cognitive functioning, reading, writing, and academic achievement. Specific subscales (e.g., auditory-linguistic processing, visual-spatial processing, reading and writing) showed expected relationships with external criteria, supporting the battery’s theoretical foundations and applied diagnostic utility.

Overall, the SB6/18 battery may be regarded as a psychometrically robust instrument for the comprehensive assessment of cognitive processes and academic skills in children and adolescents aged 6–18 years. Evidence for its strong reliability, supported factorial validity, and substantial external validity indicates that it is well suited to both clinical practice and research, particularly in the context of identifying learning difficulties and informing educational planning.

**Table 1.** Psychometric properties of the SB6/18 battery – scales and subscales.

| Scale | Reliability (Cronbach's α/test-retest) | Subscale | Test/indicator | Validity | Reliability (Cronbach's α/test-retest) |
| --- | --- | --- | --- | --- | --- |
| Visual-spatial processing | α = 0.96 | Visualisation | Visual analysis and synthesis | Strong structural agreement, correlations with TONI-4* | α = 0.86 |
|  |  | Rotation speed | Figure rotations | Correlations with fluid intelligence tests – SB5** | α = 0.90 |
|  |  | Perceptual organisation | “Gdańsk figure”–copy | Confirmed validity based on structural model | α = 0.74 |
|  |  | Visual memory | “Gdańsk figure”–memory | Confirmed validity based on structural model | α = 0.74 |
|  |  | Visuomotor coordination | “Gdańsk figure”–coordination indicators | Correlations with psychomotor coordination tests – B10/12*** | α = 0.76 |
| Auditory-linguistic processing | α = 0.97 | **Phonetic coding–linguistic aspect**  **(PC-LA)** | Syllabic and phonemic analysis, paronyms | Strong relations with speech and reading comprehension | α = 0.95 |
|  |  | **Phonetic coding–cognitive complexity**  **(PC-CC)** | Syllable inversion and spoonerisms | Associations with decoding skills – B10/12*** | α = 0.98 |
|  |  | Resistance to auditory distractors | Auditory distractors | Confirmed construct validity | α = 0.89 |
| Processing speed | 0,83^A^ | Perceptual speed | Symbols, pair cancellation | Strong correlations with information processing speed – B10/12*** | 0,73-0,88^B^ |
|  |  | Verbal information processing speed | True/false sentences–time | Consistency with SB5** outcomes | 0.83^A^ |
|  |  | **Rapid naming (RAN)** | Rapid naming | Correlations with RAN tests – B10/12*** |  |
| Long-term memory | α = 0.91 | Associative memory | Associations | Strong relations with TOMAL-2**** | α = 0.91 |
|  |  | Semantic memory | Recall | Consistency with semantic memory performance | 0.82^A^ |
| Knowledge | α = 0.98 | General Knowledge | Where? What? | Correlations with crystallised knowledge SB5** | α = 0.93 |
|  |  | Lexical Knowledge | Synonyms, Antonyms | Links with vocabulary breadth | α = 0.97 |
| Reading and Writing | α = 0.95 | **Decoding (D)** | Pseudoword | Validity regarding reading difficulties | 0.94^A^ |
|  |  | **Reading comprehension (RC)** | Letter recognition, True/false sentences, Cloze sentences, New planet | Correlations with text comprehension and phonology – B10/12*** | α = 0.94 |
|  |  |  | **Reading fluency (RF)** | Correlations with text comprehension and phonology – B10/12*** |  |
|  |  | Writing | Words, sentences, and written expression | Validity regarding writing ability | α = 0.91 |
|  |  | Orthography | Dictation | Strong links with dysorthographia diagnosis | 0.86^A^ |

* TONI-4 - Brown, L., Sherbenou, R. J., Johnsen, S. K. (2010). *Test of nonverbal intelligence* (4th ed.). Austin, TX: PRO-ED.

** SB5 - Roid, G. H., Sajewicz-Radtke, U., Radtke, B. M. & Lipowska, M. *Skale Inteligencji Stanford-Binet, Edycja Piąta* [Stanford-Binet Intelligence Scales, Fifth Edition]. Gdańsk: Pracownia Testów Psychologicznych
i Pedagogicznych [Laboratory of Psychological and Pedagogical Tests] (2017).

*** B10/12 – Bogdanowicz, M., Kalka, D., Karpińska, E., Sajewicz-Radtke, U. & Radtke, B.M. (2020). *Bateria metod diagnozy przyczyn niepowodzeń szkolnych u uczniów w wieku 10–12 lat.*

*B10/12* [A battery of methods for diagnosing the causes of school failure in students aged 10–12. B10/12]. Pracownia Testów Psychologicznych i Pedagogicznych [Laboratory of Psychological and Educational Tests].

**** TOMAL-2 - Reynolds, C. R., Voress, J. K., Sajewicz-Radtke, U., & Radtke, B. M. (2024). *Test Pamięci i Uczenia się TOMAL-2* [Test of Memory and Learning TOMAL-2]. Pracownia Testów Psychologicznych i Pedagogicznych [Laboratory of Psychological and Educational Tests].

^A^ Average reliability estimated using the test–retest method for the indicators comprising the scale

^B^ Reliability interval estimated using the test-retest method for the indicators making up the subscale.

Note: Abbreviations used in the manuscript are given in parentheses.

**Table 2.** Characteristics of SB6/18.

| 1. **VISUAL–SPATIAL PROCESSING SCALE** | This scale assesses an individual’s ability to perceive, analyze, and organize visual information and spatial relations. It measures how efficiently a person can interpret visual patterns, manipulate shapes mentally, and coordinate visual input with motor actions. | | | |  |  |
| --- | --- | --- | --- | --- | --- | --- |
| **SUBSCALE** | **TEST/INDICATOR** | | **ASSESSED FUNCTION/SKILL** | |  |  |
| Visualization | Visual analysis and synthesis | | Analyze and synthesize visual stimuli, reconstructing shapes and visual information. | | |  |
| Rotation speed | Figure rotation | | Mentally rotating geometric shapes to identify equivalent or matching figures within a 90-second time limit. | | |  |
| Perceptual organization | “Gdańsk figure”-copy | | Structuring and reproducing complex visual forms based on their spatial configuration. | | |  |
| Visual memory | “Gdańsk figure”-memory | | Recall and reproduce a previously shown visual figure. | | |  |
| Visual-motor coordination | “Gdańsk figure”-coordination indicators of reproduction accuracy | | Integrating visual input with fine motor control; accuracy and coordination during drawing or tracing tasks. | | |  |
| 1. **AUDITORY-LINGUISTIC PROCESSING SCALE** | This scale captures processes responsible for perceiving, differentiating, and manipulating auditory information, particularly those linked with phonological and linguistic development. It focuses on how efficiently an individual can recognize speech sounds, discriminate between them, and process spoken language under challenging conditions. | | | | | |
| **SUBSCALE** | **TEST/INDICATOR** | | | **ASSESSED FUNCTION/SKILL** |  |  |
| **Phonetic coding–linguistic aspect** | Syllabic and Phonemic Analysis and synthesis | | | Segmenting words into syllables or phonemes (analysis) and blending them to form full words (synthesis) |  |  |
|  | Paronym analysis | | | Segmenting words into syllables or phonemes (analysis) and blending them to form full words (synthesis) |  |  |
| **Phonetic coding–cognitive complexity** | Syllabic inversion and spoonerisms | | | Swap the positions of syllables in two-syllable words, while the “spoonerisms” part required them to exchange the initial syllables of two-word phrases. |  |  |
| Resistance to auditory distractors | Auditory distractors | | | Answer questions presented through headphones, which are obscured by white noise and randomly spoken syllables. |  |  |
| 1. **PROCESSING SPEED SCALE** | This scale evaluates the rate and fluency with which an individual can perform simple perceptual and cognitive tasks that require attention and automaticity. It reflects both visual and verbal processing efficiency. | | | |  |  |
| **SUBSCALE** | **TEST/INDICATOR** | **ASSESSED FUNCTION/SKILL** | | |  |  |
| Perceptual speed | Symbols | Rapid and accurate visual scanning, identification, and matching of patterns, within a 60-second time limit. | | |  |  |
|  | Pair crossing | Identify and cross out each instance of the specified pair “cat-clock” from a series of stimuli presented in a specific order, within a 120-second time limit. | | |  |  |
|  | **Rapid naming (RAN)** | Speed of visual information processing and lexical access, within a 60-second time limit. | | |  |  |
| Verbal processing speed | True/false sentences – time | Verbal information processing speed, within a 60-second time limit. | | |  |  |
| 1. **LONG-TERM MEMORY SCALE** | This scale focuses on how efficiently an individual can acquire, store, and retrieve information over time. It distinguishes between associative learning and semantic knowledge. | | | |  |  |
| **SUBSCALE** | **TEST/INDICATOR** | **ASSESSED FUNCTION/SKILL** | | |  |  |
| Associative memory | Associations | Capacity to remember and connect information by forming associations. | | |  |  |
| Semantic memory | Recall | Ability to retrieve information from memory after a single hearing. | | |  |  |
| 1. **KNOWLEDGE SCALE** | The Knowledge scale assesses the scope and depth of factual and linguistic information gained through life experience and formal education. It reflects both general knowledge and verbal-linguistic competence. | | | |  |  |
| **SUBSCALE** | **TEST/INDICATOR** | **ASSESSED FUNCTION/SKILL** | | |  |  |
| General knowledge | Where? What? | Extent of general knowledge. | | |  |  |
| Lexical knowledge | Synonyms, Antonyms | Knowledge of vocabulary and linguistic representations. | | |  |  |
| 1. **READING AND WRITING SCALE** | This scale measures core academic skills that are critical for literacy and learning. It integrates reading accuracy, comprehension, and written expression. | | | |  |  |
| **SUBSCALE** | **TEST/INDICATOR** | **ASSESSED FUNCTION/SKILL** | | |  |  |
| **Decoding** | Pseudowords | Ability to decode words phonetically, within a 60-second time limit. | | |  |  |
| **Reading comprehension** | Letter recognition | Proficiency in recognizing letters. | | |  |  |
|  | **Reading fluency** | Accuracy and comprehension in reading aloud, within a 60-second time limit. | | |  |  |
|  | True/false sentences | Reading comprehension and logical reasoning. | | |  |  |
|  | Gap sentences/New planet | Semantic comprehension and silent reading proficiency | | |  |  |
| Writing | Words, sentences, and written statement | Written expression skills. | | |  |  |
| Orthography | Dictation | Spelling and short-term memory. | | |  |  |

Together, above tables provide above are comprehensive overview of the SB6/18’s theoretical and operational framework, making it a valuable appendix for researchers and practitioners in educational and psychological assessment contexts. **In all the tables, the elements included in the present study have been highlighted in bold.** This formatting facilitates the identification of relevant components used for analysis and interpretation in the study’s context. The SB6/18 battery is routinely employed in psychological counselling and diagnostic services in Poland to investigate the underlying determinants of learning difficulties.
