# Supplementary material for: Identifying dyslexia-consistent reading profiles in mild intellectual disability: cluster-derived severity gradients and severity-calibrated classification rules
Source: Front Psychiatry. 2026 May 29;17:1805069. doi: 10.3389/fpsyt.2026.1805069 (PMC13260349; doi:10.3389/fpsyt.2026.1805069)
Supplement: Supplementary file 2 [file Table2.docx]

Supplementary Materials

# Sensitivity Analysis of Clustering Solutions

To examine the robustness of the clustering results, we conducted a sensitivity analysis using an alternative distance metric (Euclidean distance) and linkage method (Ward’s method). Cluster stability was evaluated using bootstrap resampling with Jaccard similarity coefficients, following the same criteria as in the primary analysis. Additionally, between-cluster differences were examined to assess the comparability of profile structure across specifications.

**Table S1.** Sensitivity Analysis: Bootstrap Stability of Hierarchical Cluster Solutions (Euclidean Distance, Ward’s Method)

| **Number of Clusters Tested** | **Stable Clusters (n)** | **Mean Jaccard** | **Median Jaccard** | **Min Jaccard** | **Max Jaccard** |
| --- | --- | --- | --- | --- | --- |
| 2 | 1 | 0.73 | 0.73 | 0.67 | 0.79 |
| 3 | 0 | 0.59 | 0.56 | 0.55 | 0.66 |
| 4 | 0 | 0.59 | 0.56 | 0.50 | 0.74 |
| 5 | 1 | 0.61 | 0.59 | 0.50 | 0.80 |

*Note.* Stable clusters are defined as clusters with a Jaccard similarity coefficient ≥ .75; values < .60 indicate substantial instability.

**Table S2.** Between-Cluster Comparisons of Reading and Cognitive–Linguistic Measures (Euclidean Distance, Ward’s Method)

| **Variable** | **Cluster 1  (n = 197)** | |  | **Cluster 2  (n = 249)** | |  | **Welch’s t-test** | | | **Cohen’s d** | |
| --- | --- | --- | --- | --- | --- | --- | --- | --- | --- | --- | --- |
|  | M | SD |  | M | SD |  | Est. | df | p | Est. | Magnitude |
| D | 1.29 | 0.64 |  | 4.35 | 1.88 |  | -24.03 | 317.66 | < .001 | -2.18 | large |
| RF | 1.26 | 0.50 |  | 3.29 | 1.45 |  | -20.57 | 317.78 | < .001 | -1.87 | large |
| RC | 1.06 | 0.29 |  | 2.29 | 1.43 |  | -13.15 | 272.78 | < .001 | -1.18 | large |
| PC–LA | 1.36 | 0.75 |  | 2.73 | 1.47 |  | -12.77 | 385.49 | < .001 | -1.18 | large |
| PC–CC | 2.32 | 0.88 |  | 3.07 | 1.34 |  | -7.12 | 430.86 | < .001 | -0.66 | moderate |
| RAN | 2.76 | 1.43 |  | 3.49 | 1.47 |  | -4.68 | 443.14 | < .001 | -0.44 | small |

*Note.* D = Decoding; RF = Reading Fluency; RC = Reading Comprehension; PC–LA = Phonetic Coding – Linguistic Aspect; PC–CC = Phonetic Coding – Cognitive Complexity; RAN = Rapid Automatized Naming.

The results of the sensitivity analysis closely mirrored those obtained in the primary clustering solution. In particular, the two-cluster solution remained the only partition without unstable clusters, and the pattern of between-cluster differences was highly comparable. Across both specifications, clusters were primarily differentiated by overall severity of reading-related difficulties, with the largest effects observed for decoding and reading fluency, followed by reading comprehension and phonological measures, and smaller effects for RAN.

These findings indicate that the identified cluster structure is robust to alternative distance metrics and linkage methods and reflects a stable severity-based organisation of reading and cognitive–linguistic performance in individuals with mild intellectual disability.
